# Teaching an Old Dog New Tricks for Achieving CB_2_-Selective Inverse Agonism

**DOI:** 10.1021/acscentsci.4c00673

**Published:** 2024-05-08

**Authors:** Morgane Mando, Christopher W. Cunningham, Alexander J. Grenning

**Affiliations:** † Department of Chemistry, University of Minnesota, Minneapolis, Minnesota 55455, United States; ‡ Department of Pharmaceutical Sciences, Concordia University Wisconsin, Mequon, Wisconsin 53097, United States

The endocannabinoid signaling system (ECSS) is a “master regulator” of diverse physiologic processes throughout the body. As such, modulating the ECSS has broad therapeutic potential, from treating pain, stress, and anxiety to osteoporosis, neurodegenerative disease, and perhaps even cancer. Despite its potential as a therapeutic target, the expression and function of the CB_2_ cannabinoid receptor (CB_2_R) in the ECSS function remain elusive. Where is it (?), what does it do (?), and is it druggable (?) are still “big questions” on the table!^1^ And versions of these questions are old. Endocannabinoid research dates back to the 1940s, well before there was any knowledge of the endocannabinoid system. Phytocannabinoids, natural products that we now know target the ECSS, were being isolated, elucidated, synthesized, and studied for therapeutic potential at this time. Furthermore, some of this early research was carried out by none other than organic chemistry giant Dr. Roger Adams (1889–1971) and his research team, who can be credited with the first total synthesis of a phytocannabinoid.^2^ Large contributions to cannabinoid research must also be linked to the late great Dr. Raphael Mechoulam (1930–2023), who had a storied career in the field. Among the many important molecules that his team at Hebrew University developed is HU-308, a cannabinoid receptor 2 (CB_2_R)-selective agonist.^3^ In this issue of *ACS Central Science*, we witness firsthand how research synergy among computational chemists, synthetic chemists, chemical biologists, and pharmacologists can yield profound new findings for endocannabinoid system research possible only through collaboration. In this case, it is teaching the old dog (HU-308) a valuable new trick: to function as a CB_2_R inverse agonist and potentially address some of these aforementioned big questions!

**Figure 1:**
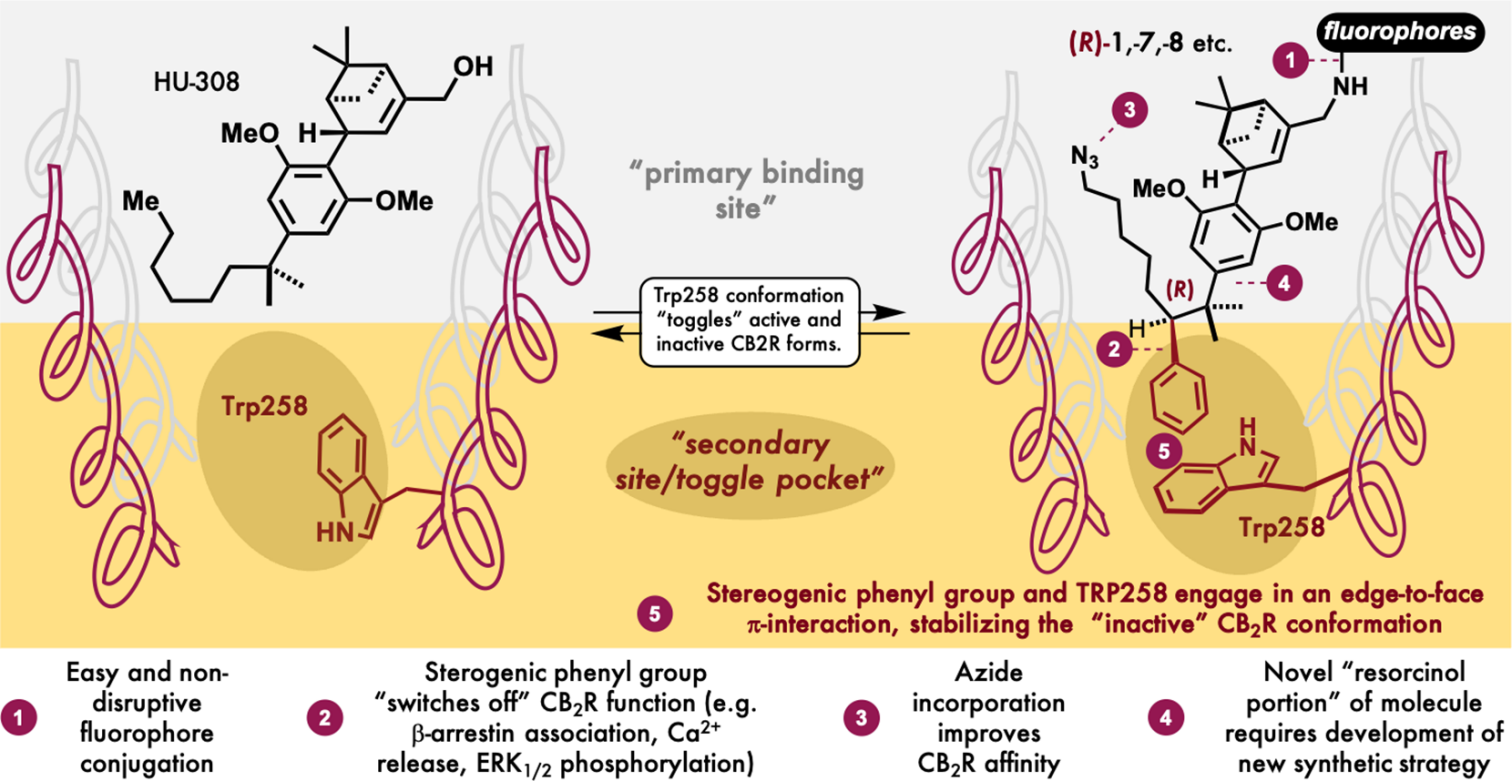
Illustration of active (left) and inactive (right) conformations of CB_2_R.

To best appreciate this scientific work, it is important to first discuss some basic pharmacology. When an agonist binds a receptor, it stabilizes that receptor in its active state, which can be thought as “switching it on.” Conversely, an inverse agonist stabilizes a constitutively active receptor in its inactive state, thereby “switching off” its ability to promote a signal. When a receptor is activated by an agonist, intracellular processes can be triggered that may interfere with receptor expression and function: pathway uncoupling, receptor internalization, and receptor degradation can all occur when an agonist probe is used. This is why an inverse agonist profile is most desirable in a fluorescent probe: binding to the receptor is unlikely to disrupt its expression at the cell surface. With the goal in mind of turning a high-selectivity CB_2_R agonist into an inverse agonist, the team focused on a region of the receptor responsible for controlling the active/inactive equilibrium (Figure 1). More specifically, one residue, TRP258^6.48^, serves as the toggle switch for CB_2_R (and 78% of other G protein-coupled receptors, GPCRs) activation/inactivation depending on its conformation. Indeed, useful molecular probes that retain their inverse agonist activity and affinity are still in need of discovery to broaden endocannabinoid system understanding and therapeutic development. Considering CB_2_R’s role in modulating pain, precision control of function here is of timely relevance as we continue to respond to the opioid crisis.

The strategy of turning HU-308 from an agonist to an inverse agonist is elegant in its simplicity. The team took inspiration from two recent crystal structures of CB_2_R in active^4^ and inactive^5^ conformations when HU-308 and AM10257 are bound, respectively. Noting that a phenyl ring of AM10257 prevents the toggle switch from reaching an active state conformation, a “hybrid” was designed that incorporated a stereogenic 2′-phenyl group into the dimethylheptyl tail of HU-308. The stereogenic 2′-phenyl group proved to be a double-edged sword: molecular docking suggested that the resulting compound **1** would be an inverse agonist, but including this group complicates de novo chemical synthesis. The team devised a racemic, chiral separation-based synthesis for these novel probes including some synthetic flexibility for attaching additional functional groups at molecular termini to improve affinity and selectivity as well as for the attachment of fluorescent probes. Excitingly, the parent structures (*S*)-**1** and (*R*)-**1** display chirality-specific (in favor of the *R* enantiomer) and low nanomolar affinity and selectivity at CB_2_R. And with a stroke of luck, the addition of the fluorescent probe to the parent ligand does not disrupt the ligand affinity, efficacy, and selectivity!

That the new scaffold (*R*)-**1** retains its desirable pharmacodynamic properties when complexed to different fluorescent probes is particularly interesting and useful. Fluorescent probes themselves substantially impact the molecular weight, hydrogen bonding, and ionic properties of the probe, which can interfere with probe function.^6,7^ Indeed, compounds (*R*)-**7** and (*R*)-**9** have attached dyes that have multiple weakly acidic and basic functional groups that are ionized at physiological pH. This could have disrupted ligand binding due to potential incompatibilities with the hydrophobic nature of the CB_2_R binding site. The unpredictable nature of attaching a dye to a probe was previously reported by another group.^8^ In their series, attaching different dyes and linkers to the chromenepyrazole pharmacophore resulted in agonists as well as inverse agonists. That scaffold **1** resulted exclusively in CB_2_R inverse agonists suggests that this pharmacophore operates in a different way and could be amenable to many more modifications.

It remains to be seen whether the ultimate comment in the manuscript will be realized, whether “the strategy and experimental framework disclosed herein may aid in the structure-based design of agonists, antagonists, and inverse agonists for GPCRs beyond CB_2_R.” There are many different types of toggle switch amino acid sequences—CWxP here, while others include NPxxY, D/ERY, etc.—and influencing remote switches directly by ligands can also be a challenge.^9^ Nonetheless, this study demonstrates the value of collaboration and the importance of finding new molecular scaffolds^10^ for further probing and understanding the complex endocannabinoid system. Without a doubt, switching off the CB_2_R function will switch on creative science muscles around the world, encouraging more collaborative teams to assemble to achieve endocannabinoid system understanding and drug discovery.
